# Supplementary material for: A Metabolism-Related Gene Prognostic Index for Prediction of Response to Immunotherapy in Lung Adenocarcinoma
Source: Int J Mol Sci. 2022 Oct 12;23(20):12143. doi: 10.3390/ijms232012143 (PMC9602971; doi:10.3390/ijms232012143)
Supplement: Supplementary file 1 [file ijms-23-12143-s001.zip › Table S1.pdf]

Table S1. List of 72 genes used for NMF analysis

Gene ID	HR	HR.95L	HR.95H	*P*-value
SLC2A1	1.337703	1.17351	1.52487	1.33×10^{-05}
TYMS	1.302313	1.133885	1.49576	0.000185
GAPDH	1.690166	1.355751	2.107069	3.08×10^{-06}
TPI1	1.624025	1.252982	2.104945	0.000248
PKM	1.699031	1.294987	2.229139	0.00013
CFTR	0.770124	0.660065	0.898534	0.000901
PFKP	1.296363	1.129827	1.487446	0.000216
CDKN3	1.357364	1.17077	1.573695	5.13×10^{-05}
HMGA1	1.327329	1.159703	1.519183	3.94×10^{-05}
GPI	1.505531	1.212517	1.869355	0.000211
RAD51	1.361998	1.145176	1.61987	0.000479
SELENBP1	0.833451	0.749218	0.927153	0.000804
TK1	1.347741	1.164589	1.559697	6.21×10^{-05}
PAICS	1.428041	1.168826	1.744742	0.00049
TFAP2A	1.256636	1.105831	1.428006	0.000461
HMMR	1.407814	1.210083	1.637854	9.45×10^{-06}
CDK1	1.283304	1.123426	1.465935	0.000238
FANCI	1.301766	1.120274	1.512662	0.000576
FEN1	1.42669	1.165886	1.745834	0.00056
ARNTL2	1.374399	1.20538	1.567119	2.03×10^{-06}
RRM2	1.323105	1.165783	1.501657	1.46×10^{-05}
FOXM1	1.243634	1.116179	1.385644	7.74×10^{-05}
BIRC5	1.264114	1.105892	1.444973	0.000592
AURKA	1.276108	1.107286	1.470669	0.000758
CCNA2	1.34128	1.167933	1.540355	3.20×10^{-05}
IGF2BP3	1.185289	1.071358	1.311337	0.000978
CDX2	1.459333	1.206824	1.764675	9.65×10^{-05}
PLK1	1.406757	1.217405	1.62556	3.71×10^{-06}
MKI67	1.36455	1.160958	1.603846	0.000163
KPNA2	1.424799	1.18977	1.706256	0.000119
GGTLC1	0.825819	0.741522	0.9197	0.000495
HMGA2	1.20183	1.08671	1.329145	0.000345
CCT6A	1.485202	1.236351	1.784142	2.36×10^{-05}
PKMYT1	1.395169	1.146681	1.697506	0.000876
CCNB1	1.33684	1.168922	1.528879	2.24×10^{-05}
PTTG1	1.343444	1.143892	1.577807	0.00032
UBE2S	1.270829	1.114907	1.448556	0.000332
CDC25C	1.550146	1.256819	1.911933	4.21×10^{-05}
EIF3B	1.578799	1.20303	2.07194	0.000992
CENPF	1.267328	1.103083	1.456028	0.000822
EXO1	1.407629	1.206433	1.642377	1.39×10^{-05}

SPC25	1.364324	1.162458	1.601244	0.000143
CDC20	1.189893	1.075457	1.316506	0.000752
ASPM	1.227216	1.103134	1.365255	0.000167
LYPD3	1.236474	1.131892	1.35072	2.51×10^{-06}
FKBP4	1.545077	1.278105	1.867814	6.95×10^{-06}
PKP2	1.389498	1.223867	1.577544	3.79×10^{-07}
BCAN	1.516268	1.200843	1.914547	0.000469
NEIL3	1.284988	1.118872	1.475767	0.000385
CKS1B	1.338641	1.13781	1.574919	0.000437
ABCA3	0.84763	0.772775	0.929737	0.000458
LDHA	1.990925	1.53424	2.583547	2.22×10^{-07}
F2	1.209459	1.082727	1.351025	0.000759
ALDOA	1.535959	1.209518	1.950505	0.000431
ANGPTL4	1.197642	1.091004	1.314703	0.00015
ABCC2	1.159944	1.084812	1.240279	1.41×10^{-05}
CYP4B1	0.867625	0.81192	0.927151	2.74×10^{-05}
SFTPD	0.908937	0.858744	0.962063	0.000986
NPAS2	1.490016	1.225973	1.810929	6.15×10^{-05}
CACNA2D2	0.847798	0.775074	0.927346	0.000308
KRT8	1.513487	1.25215	1.829368	1.83×10^{-05}
KRT18	1.511776	1.251879	1.82563	1.75×10^{-05}
SMOX	1.353578	1.16089	1.578248	0.000112
CKAP4	1.497898	1.187785	1.888978	0.00064
ARRB1	0.706487	0.581606	0.858183	0.000463
PRSS3	1.20465	1.085005	1.337489	0.000486
CRTAC1	0.80888	0.715563	0.914366	0.000695
GRIA1	0.520238	0.358448	0.755053	0.000585
B4GALT4	1.426044	1.167697	1.741549	0.000501
KRT6A	1.164779	1.09872	1.23481	3.05×10^{-07}
LIFR	0.747381	0.637647	0.876001	0.000326
DPYSL2	0.731915	0.623681	0.858932	0.000132
