# Supplementary material for: A Metabolism-Related Gene Prognostic Index for Prediction of Response to Immunotherapy in Lung Adenocarcinoma
Source: Int J Mol Sci. 2022 Oct 12;23(20):12143. doi: 10.3390/ijms232012143 (PMC9602971; doi:10.3390/ijms232012143)
Supplement: Supplementary file 1 [file ijms-23-12143-s001.zip › Table S2.pdf]

Table S2. Clinical information of TCGA cohort

Variable		MRG-high (n = 250)	MRG-low (n = 250)	*P*-value
Age	≥66	117(48%)	135(56%)	0.164
	< 65	128(52%)	108(44%)	
Gender	Female	120(48%)	150(60%)	0.009
	Male	130(52%)	100(40%)	
Distant metastasis	M0	165(67%)	167(67%)	0.905
	M1	13(5%)	11(4%)	
	MX	69(28%)	71(29%)	
Regional lymph nodes	N0	149(60%)	175(70%)	0.079
	N1	55(22%)	39(16%)	
	N2	39(16%)	30(12%)	
	N3	2(1%)	0(0%)	
	NX	4(2%)	6(2%)	
Tumor stage	Stage I	115(47%)	153(62%)	0.007
	Stage II	71(29%)	48(20%)	
	Stage III	46(19%)	34(14%)	
	Stage IV	14(6%)	11(4%)	
Primary tumor	T 1	71(28%)	96(38%)	0.2
	T 2	143(57%)	124(50%)	
	T 3	25(10%)	20(8%)	
	T 4	9(4%)	9(4%)	
	T X	2(1%)	1(0%)	
Survival status	Alive	142(57%)	176(70%)	0.002
	Dead	108(43%)	74(30%)	
