# Supplementary material for: A Metabolism-Related Gene Prognostic Index for Prediction of Response to Immunotherapy in Lung Adenocarcinoma
Source: Int J Mol Sci. 2022 Oct 12;23(20):12143. doi: 10.3390/ijms232012143 (PMC9602971; doi:10.3390/ijms232012143)
Supplement: Supplementary file 1 [file ijms-23-12143-s001.zip › Table S3.pdf]

Table S3. Detailed data on GSEA analysis of TCGA MRG subgroups

ID	Enrichment score	NES	*P*-value	*P*.adjust
KEGG_CELL_CYCLE	0.703911286	2.592899864	1.00×10^{-10}	1.84×10^{-8}
KEGG_DNA_REPLICATION	0.757108667	2.218431307	1.57×10^{-6}	0.000144071
KEGG_SPLICEOSOME	0.53070727	1.968987763	3.82×10^{-6}	0.00023437
KEGG_OOCYTE_MEIOSIS	0.56536538	2.004376743	1.49×10^{-5}	0.000686194
KEGG_SYSTEMIC_LUPUS_ERYTHEMATOSUS	0.534834865	1.927804298	2.24×10^{-5}	0.000824956
KEGG_ASTHMA	-0.781699305	-2.007590278	3.13×10^{-5}	0.00095854
KEGG_PROTEASOME	0.651067224	2.010354982	0.000186927	0.004913518
KEGG_P53_SIGNALING_PATHWAY	0.565815999	1.896765453	0.000276954	0.00636994
KEGG_MISMATCH_REPAIR	0.726295169	1.956867861	0.000771868	0.015780409
KEGG_HOMOLOGOUS_RECOMBINATION	0.688952263	1.940342797	0.001004316	0.016875488
KEGG_PROGESTERONE_MEDIATED_OOCYTE_MATURATION	0.534730471	1.81566744	0.001089985	0.016875488
KEGG_ECM_RECEPTOR_INTERACTION	0.52006321	1.762332897	0.001100575	0.016875488
KEGG_SMALL_CELL_LUNG_CANCER	0.498327834	1.707715459	0.001332201	0.01885577
KEGG_INTESTINAL_IMMUNE_NETWORK_FOR_IGA_PRODUCTION	-0.603535824	-1.794408479	0.002027265	0.026644049
KEGG_PATHWAYS_IN_CANCER	0.349778265	1.421836743	0.002374366	0.029125555
KEGG_PYRIMIDINE_METABOLISM	0.448521074	1.585958075	0.003820142	0.043931631
KEGG_MELANOMA	0.519541195	1.677988302	0.006616399	0.071612784
KEGG_FOCAL_ADHESION	0.363289754	1.394735251	0.012593186	0.128730344
KEGG_VIRAL_MYOCARDITIS	-0.475658927	-1.557048802	0.015970956	0.149426937
KEGG_BASE_EXCISION_REPAIR	0.555958411	1.625940329	0.016242058	0.149426937
KEGG_REGULATION_OF_ACTIN_CYTOSKELETON	0.356113135	1.363416678	0.017972447	0.153590761
KEGG_CITRATE_CYCLE_TCA_CYCLE	0.579206705	1.644208568	0.018364113	0.153590761
KEGG_NEUROACTIVE_LIGAND_RECEPTOR_INTERACTION	0.397299196	1.430496072	0.019277718	0.154221745
KEGG_RNA_DEGRADATION	0.478038859	1.548688235	0.020583665	0.157295335
KEGG_AMINOACYL_TRNA_BIOSYNTHESIS	0.523600756	1.605032395	0.021739377	0.157295335
KEGG_PATHOGENIC_ESCHERICHIA_COLI_INFECTION	0.480519902	1.521256167	0.022642439	0.157295335
KEGG_NUCLEOTIDE_EXCISION_REPAIR	0.519663542	1.60460879	0.023081381	0.157295335
KEGG_DRUG_METABOLISM_OTHER_ENZYMES	0.575149495	1.638807521	0.028155499	0.18502185
KEGG_UBIQUITIN_MEDIATED_PROTEOLYSIS	0.378439755	1.406633492	0.030752587	0.195119864
KEGG_FC_EPSILON_RI_SIGNALING_PATHWAY	-0.410875259	-1.367060244	0.046728972	0.281097597
KEGG_ALLOGRAFT_REJECTION	-0.524213183	-1.482498273	0.047358834	0.281097597

Table S3. Detailed data on GSEA analysis of GSE68465 MRG subgroups

ID	Enrichment score	NES	*P*-value	*P*.adjust
KEGG_CELL_CYCLE	0.66832875	2.528008252	1.00×10^{-10}	1.83×10^{-8}
KEGG_SPLICEOSOME	0.552586013	2.038039936	1.60×10^{-6}	0.000146233
KEGG_PROTEASOME	0.717440865	2.25680462	2.59×10^{-6}	0.000157886
KEGG_DNA_REPLICATION	0.731683489	2.227160948	3.56×10^{-6}	0.000162994
KEGG_PYRIMIDINE_METABOLISM	0.53230305	1.858454927	0.00026267	0.009614049
KEGG_PARKINSONS_DISEASE	0.500204419	1.800510314	0.00071959	0.018812195
KEGG_OXIDATIVE_PHOSPHORYLATION	0.501229403	1.792179479	0.00063811	0.018812195
KEGG_P53_SIGNALING_PATHWAY	0.538212425	1.802692269	0.00087925	0.019828053
KEGG_OOCYTE_MEIOSIS	0.481200675	1.748645875	0.00097515	0.019828053
KEGG_MISMATCH_REPAIR	0.68647668	1.895399924	0.00202624	0.033709342
KEGG_DRUG_METABOLISM_CYTOCHROME_P450	-0.520644276	-1.79334852	0.00191585	0.033709342
KEGG_NUCLEOTIDE_EXCISION_REPAIR	0.542513232	1.73701444	0.00377149	0.05554313
KEGG_GLYCEROPHOSPHOLIPID_METABOLISM	-0.480423407	-1.68136871	0.00394568	0.05554313
KEGG_HOMOLOGOUS_RECOMBINATION	0.630591674	1.81560194	0.00438307	0.057293096
KEGG_VASCULAR_SMOOTH_MUSCLE_CONTRACTION	-0.422099755	-1.62511204	0.00500142	0.061017416
KEGG_PRIMARY_BILE_ACID_BIOSYNTHESIS	-0.727729244	-1.86394231	0.00623629	0.06426892
KEGG_BASE_EXCISION_REPAIR	0.579648412	1.717530952	0.00688472	0.06426892
KEGG_COMPLEMENT_AND_COAGULATION_CASCADES	-0.457117279	-1.64120290	0.00602428	0.06426892
KEGG_VIRAL_MYOCARDITIS	-0.452060575	-1.61927854	0.00673733	0.06426892
KEGG_NEUROACTIVE_LIGAND_RECEPTOR_INTERACTION	-0.31338204	-1.37700661	0.00702392	0.06426892
KEGG_PPAR_SIGNALING_PATHWAY	-0.452794016	-1.60193726	0.00979001	0.085313013
KEGG_CITRATE_CYCLE_TCA_CYCLE	0.563825309	1.670646206	0.01097455	0.091288367
KEGG_FATTY_ACID_METABOLISM	-0.507833418	-1.65151733	0.01340825	0.098148397
KEGG_RNA_DEGRADATION	0.507454392	1.632771279	0.01322218	0.098148397
KEGG_LYSOSOME	-0.369348259	-1.44329290	0.01257482	0.098148397
KEGG_NITROGEN_METABOLISM	-0.588371683	-1.68466010	0.01639330	0.107528217
KEGG_ARACHIDONIC_ACID_METABOLISM	-0.478119667	-1.60548892	0.01555500	0.107528217
KEGG_AMYOTROPHIC_LATERAL_SCLEROSIS_ALS	0.471705399	1.543708225	0.01799081	0.107528217
KEGG_HYPERTROPHIC_CARDIOMYOPATHY_HCM	-0.403924467	-1.49333244	0.01751435	0.107528217
KEGG_UBIQUITIN_MEDIATED_PROTEOLYSIS	0.394689017	1.488479172	0.01791631	0.107528217
KEGG_MAPK_SIGNALING_PATHWAY	-0.305860926	-1.34658990	0.01821516	0.107528217

Table S3. Detailed data on GSEA analysis of GSE31210 MRG subgroups

ID	Enrichment score	NES	*P*-value	*P*.adjust
KEGG_CELL_CYCLE	0.683687359	2.440632349	1.00×10^{-10}	1.84×10^{-8}
KEGG_CYTOKINE_CYTOKINE_RECEPTOR_INTERACTION	0.511844551	1.96103749	2.45×10^{-8}	2.26×10^{-6}
KEGG_DNA_REPLICATION	0.72544663	2.101106771	2.12×10^{-5}	0.001298063
KEGG_CHEMOKINE_SIGNALING_PATHWAY	0.481004302	1.780589631	3.20×10^{-5}	0.001471231
KEGG_PROTEASOME	0.681365785	2.001714868	6.04×10^{-5}	0.001741314
KEGG_DRUG_METABOLISM_CYTOCHROME_P450	-0.551411827	-1.97258211	7.0×10^{-5}	0.001741314
KEGG_ANTIGEN_PROCESSING_AND_PRESENTATION	0.588440152	1.922303009	4.76×10^{-5}	0.001741314
KEGG_PYRIMIDINE_METABOLISM	0.55183263	1.864975228	7.57×10^{-5}	0.001741314
KEGG_METABOLISM_OF_XENOBIOTICS_BY_CYTOCHROME_P450	-0.561561721	-1.97795877	9.04×10^{-5}	0.001742724
KEGG_PRIMARY_IMMUNODEFICIENCY	0.68823861	1.975419433	9.47×10^{-5}	0.001742724
KEGG_P53_SIGNALING_PATHWAY	0.579939811	1.849019763	0.00014853	0.002294511
KEGG_OOCYTE_MEIOSIS	0.517096299	1.808241746	0.00014964	0.002294511
KEGG_MISMATCH_REPAIR	0.757439064	1.952164939	0.00019692	0.002787304
KEGG_ECM_RECEPTOR_INTERACTION	0.525021041	1.750877568	0.00081055	0.009942752
KEGG_NATURAL_KILLER_CELL_MEDIATED_CYTOTOXICITY	0.474283149	1.697048953	0.00080204	0.009942752
KEGG_NOD_LIKE_RECEPTOR_SIGNALING_PATHWAY	0.554217158	1.741948191	0.00171246	0.019693346
KEGG_RETINOL_METABOLISM	-0.516965083	-1.76373387	0.00211089	0.022847312
KEGG_FATTY_ACID_METABOLISM	-0.522541148	-1.74678837	0.00262208	0.025392788
KEGG_GRAFT_VERSUS_HOST_DISEASE	0.606904801	1.741970765	0.00250589	0.025392788
KEGG_PENTOSE_PHOSPHATE_PATHWAY	0.679206575	1.820304669	0.00298766	0.02748655
KEGG_HOMOLOGOUS_RECOMBINATION	0.665970733	1.784832006	0.00487305	0.042697222
KEGG_BASE_EXCISION_REPAIR	0.613513467	1.716547969	0.00576725	0.048235215
KEGG_LEISHMANIA_INFECTION	0.483973887	1.552583589	0.00745501	0.059640154
KEGG_TOLL_LIKE_RECEPTOR_SIGNALING_PATHWAY	0.459068052	1.578559136	0.00853921	0.065467286
KEGG_FC_GAMMA_R_MEDIATED_PHAGOCYTOSIS	0.456612006	1.541272807	0.00890828	0.065564996
KEGG_VASCULAR_SMOOTH_MUSCLE_CONTRACTION	-0.389542557	-1.51364896	0.00998066	0.070632426
KEGG_DILATED_CARDIOMYOPATHY	-0.388504236	-1.48414097	0.01047399	0.071378357
KEGG_BLADDER_CANCER	0.54549217	1.604043116	0.01242718	0.081664344
KEGG_CITRATE_CYCLE_TCA_CYCLE	0.580595193	1.61922204	0.01347713	0.085510091
KEGG_WNT_SIGNALING_PATHWAY	-0.331896316	-1.33820717	0.02109897	0.129407039
KEGG_RIBOFLAVIN_METABOLISM	0.6758993	1.58953022	0.02234743	0.132642811

Table S3. Detailed data on GSEA analysis of GSE50081 MRG subgroups

ID	Enrichment score	NES	*P*-value	*P*.adjust
KEGG_CYTOKINE_CYTOKINE_RECEPTOR_INTERACTION	0.529428331	2.363568869	1.00×10^{-10}	1.84×10^{-8}
KEGG_GRAFT_VERSUS_HOST_DISEASE	0.749563407	2.404347474	9.06×10^{-9}	5.56×10^{-7}
KEGG_CHEMOKINE_SIGNALING_PATHWAY	0.4965547	2.124210355	6.09×10^{-9}	5.56×10^{-7}
KEGG_TOLL_LIKE_RECEPTOR_SIGNALING_PATHWAY	0.553779313	2.136836446	1.08×10^{-7}	4.97×10^{-6}
KEGG_ANTIGEN_PROCESSING_AND_PRESENTATION	0.584024091	2.159257191	4.14×10^{-7}	1.46×10^{-5}
KEGG_CELL_CYCLE	0.50671887	2.020615359	4.76×10^{-7}	1.46×10^{-5}
KEGG_NATURAL_KILLER_CELL_MEDIATED_CYTOTOXICITY	0.458287054	1.845283774	1.35×10^{-5}	0.000353677
KEGG_PRIMARY_IMMUNODEFICIENCY	0.628979463	2.017554711	4.87×10^{-5}	0.001120748
KEGG_TYPE_I_DIABETES_MELLITUS	0.633455869	2.077017265	6.79×10^{-5}	0.001388856
KEGG_LEISHMANIA_INFECTION	0.519389742	1.878037739	0.00012709	0.002338529
KEGG_NOD_LIKE_RECEPTOR_SIGNALING_PATHWAY	0.535568257	1.887669217	0.00015771	0.002418322
KEGG_NEUROACTIVE_LIGAND_RECEPTOR_INTERACTION	0.353923727	1.587136899	0.00014480	0.002418322
KEGG_ALLOGRAFT_REJECTION	0.621865679	1.962809942	0.00035549	0.005031667
KEGG_AUTOIMMUNE_THYROID_DISEASE	0.56512678	1.922360238	0.00054473	0.007159383
KEGG_DNA_REPLICATION	0.598740281	1.933635521	0.00060146	0.007377965
KEGG_CYTOSOLIC_DNA_SENSING_PATHWAY	0.534003885	1.858879789	0.00078734	0.009054405
KEGG_HEMATOPOIETIC_CELL_LINEAGE	0.432663725	1.639223084	0.00229221	0.024809897
KEGG_OLFACTORY_TRANSDUCTION	0.400324108	1.572810769	0.00251892	0.025749007
KEGG_ECM_RECEPTOR_INTERACTION	0.444142135	1.672485837	0.00324008	0.029808806
KEGG_P53_SIGNALING_PATHWAY	0.452653972	1.633389617	0.00322361	0.029808806
KEGG_DRUG_METABOLISM_CYTOCHROME_P450	-0.502895941	-1.72024873	0.00354733	0.031081397
KEGG_INTESTINAL_IMMUNE_NETWORK_FOR_IGA_PRODUCTION	0.504285944	1.703917901	0.00397158	0.033216922
KEGG_SELENOAMINO_ACID_METABOLISM	-0.597839383	-1.71466946	0.00666488	0.053319049
KEGG_FOCAL_ADHESION	0.326078087	1.401269548	0.008313	0.063733004
KEGG_JAK_STAT_SIGNALING_PATHWAY	0.343907434	1.432716104	0.00895094	0.065878935
KEGG_PROXIMAL_TUBULE_BICARBONATE_RECLAMATION	-0.617513679	-1.72548727	0.01106885	0.069664229
KEGG_PROPANOATE_METABOLISM	-0.546078313	-1.64921165	0.01135829	0.069664229
KEGG_FATTY_ACID_METABOLISM	-0.519558988	-1.64416313	0.01089810	0.069664229
KEGG_HOMOLOGOUS_RECOMBINATION	0.553763541	1.640501432	0.01048946	0.069664229
KEGG_OOCYTE_MEIOSIS	0.376783932	1.465837121	0.01124104	0.069664229
KEGG_MISMATCH_REPAIR	0.577641773	1.643586088	0.01300030	0.07475178
