# Supplementary material for: A Metabolism-Related Gene Prognostic Index for Prediction of Response to Immunotherapy in Lung Adenocarcinoma
Source: Int J Mol Sci. 2022 Oct 12;23(20):12143. doi: 10.3390/ijms232012143 (PMC9602971; doi:10.3390/ijms232012143)
Supplement: Supplementary file 1 [file ijms-23-12143-s001.zip › Table S4.pdf]

Table S4. Detailed data on GSVA analysis of MRG subgroups

ID	logFC	AveExpr	t	*P*-value × 10	adj.*P*.Val	B
KEGG_CELL_CYCLE	-0.225207611	-0.045163921	-17.2359039	4.00×10^{-60}	7.33×10^{-58}	125.9456841
KEGG_DNA_REPLICATION	-0.317298913	-0.051967277	-15.77397323	1.98×10^{-51}	1.82×10^{-49}	106.0796504
KEGG_HOMOLOGOUS_RECOMBINATION	-0.240618871	-0.03969603	-14.41653994	7.98×10^{-44}	4.87×10^{-42}	88.71503481
KEGG_MISMATCH_REPAIR	-0.236981035	-0.045701335	-13.15952469	3.18×10^{-37}	1.46×10^{-35}	73.65368516
KEGG_FATTY_ACID_METABOLISM	0.185984648	0.002429009	12.68049048	7.93×10^{-35}	2.90×10^{-33}	68.18900362
KEGG_PROTEASOME	-0.234924809	-0.035642788	-12.30030645	5.65×10^{-33}	1.72×10^{-31}	63.9645967
KEGG_BASE_EXCISION_REPAIR	-0.193689727	-0.027352509	-12.18054839	2.12×10^{-32}	5.02×10^{-31}	62.65493467
KEGG_PRIMARY_BILE_ACID_BIOSYNTHESIS	0.181022337	0.033351227	12.17745842	2.20×10^{-32}	5.02×10^{-31}	62.6212777
KEGG_DRUG_METABOLISM_CYTOCHROME_P450	0.146083947	0.028841926	10.99736978	5.80×10^{-27}	1.06×10^{-25}	50.27406765
KEGG_NUCLEOTIDE_EXCISION_REPAIR	-0.147924218	-0.034548422	-10.81772909	3.54×10^{-26}	5.90×10^{-25}	48.48527264
KEGG_HISTIDINE_METABOLISM	0.141129397	0.021983192	10.61525126	2.65×10^{-25}	3.73×10^{-24}	46.49859028
KEGG_PYRIMIDINE_METABOLISM	-0.131300778	-0.022236469	-10.30465471	5.45×10^{-24}	7.12×10^{-23}	43.51254164
KEGG_VALINE_LEUCINE_AND_ISOLEUCINE_DEGRADATION	0.153478623	-0.007471823	10.04483988	6.45×10^{-23}	7.87×10^{-22}	41.07258011
KEGG_BASAL_TRANSCRIPTION_FACTORS	-0.125528537	-0.027725673	-9.929554774	1.90×10^{-22}	2.17×10^{-21}	40.00698728
KEGG_OTHER_GLYCAN_DEGRADATION	0.175800955	0.019427585	9.890367118	2.74×10^{-22}	2.94×10^{-21}	39.64717574
KEGG_P53_SIGNALING_PATHWAY	-0.100263498	-0.022502686	-9.501342592	9.57×10^{-21}	9.73×10^{-20}	36.14194905
KEGG_ONE_CARBON_POOL_BY_FOLATE	-0.152203061	-0.017954202	-9.443422851	1.61×10^{-20}	1.55×10^{-19}	35.63051451
KEGG_CIRCADIAN_RHYTHM_MAMMAL	0.141519034	0.007701052	8.955475119	1.14×10^{-18}	1.04×10^{-17}	31.43077148
KEGG_SPLICEOSOME	-0.116852261	-0.035182057	-8.608683432	2.10×10^{-17}	1.83×10^{-16}	28.56573926
KEGG_TYROSINE_METABOLISM	0.101960565	0.015974197	8.354365901	1.67×10^{-16}	1.27×10^{-15}	26.52887523
KEGG_RNA_DEGRADATION	-0.110027214	-0.031188691	-8.246568899	3.94×10^{-16}	2.89×10^{-15}	25.68205594

KEGG_METABOLISM_OF_XENOBIOTICS_BY_CYTOCHROME_P450	0.110526159	0.023576746	8.131884881	9.76×10^{-16}	6.87×10^{-15}	24.79201236
KEGG_GLYOXYLATE_AND_DICARBOXYLATE_METABOLISM	-0.131759846	-0.015322131	-7.953789533	3.90×10^{-15}	2.46×10^{-14}	23.43218524
KEGG_BUTANOATE_METABOLISM	0.108293295	0.001410922	7.912051019	5.37×10^{-15}	3.28×10^{-14}	23.11744302
KEGG_GLYCOSAMINOGLYCAN_BIOSYNTHESIS_KERATAN_SULFATE	-0.123290563	-0.001048055	-7.841212415	9.23×10^{-15}	5.28×10^{-14}	22.58670561
KEGG_PENTOSE_PHOSPHATE_PATHWAY	-0.106393055	-0.023826327	-7.731243043	2.12×10^{-14}	1.18×10^{-13}	21.77139979
KEGG_RIBOFLAVIN_METABOLISM	-0.108296032	-0.006994459	-7.598857218	5.69×10^{-14}	2.89×10^{-13}	20.80384056
KEGG_PROTEIN_EXPORT	-0.12085673	-0.017445634	-6.757424103	2.11×10^{-11}	8.59×10^{-11}	15.01458434
KEGG_ASCORBATE_AND_ALDARATE_METABOLISM	0.111771933	0.001745669	6.497924542	1.16×10^{-10}	4.41×10^{-10}	13.3565485
KEGG_NON_HOMOLOGOUS_END_JOINING	-0.11295284	-0.02409945	-6.309871893	3.82×10^{-10}	1.34×10^{-09}	12.19304269
KEGG_CELL_CYCLE	-0.2252076	-0.04516392	-17.2359039	4.00×10^{-60}	7.33×10^{-58}	125.9456841
KEGG_DNA_REPLICATION	-0.317298913	-0.051967277	-15.77397323	1.98×10^{-51}	1.82×10^{-49}	106.0796504
KEGG_HOMOLOGOUS_RECOMBINATION	-0.240618871	-0.03969603	-14.41653994	7.98×10^{-44}	4.87×10^{-42}	88.71503481
KEGG_MISMATCH_REPAIR	-0.236981035	-0.045701335	-13.15952469	3.18×10^{-37}	1.46×10^{-35}	73.65368516
KEGG_FATTY_ACID_METABOLISM	0.185984648	0.002429009	12.68049048	7.93×10^{-35}	2.90×10^{-33}	68.18900362
KEGG_PROTEASOME	-0.234924809	-0.035642788	-12.30030645	5.65×10^{-33}	1.72×10^{-31}	63.9645967
KEGG_BASE_EXCISION_REPAIR	-0.193689727	-0.027352509	-12.18054839	2.12×10^{-32}	5.02×10^{-31}	62.65493467
KEGG_PRIMARY_BILE_ACID_BIOSYNTHESIS	0.181022337	0.033351227	12.17745842	2.20×10^{-32}	5.02×10^{-31}	62.6212777
KEGG_DRUG_METABOLISM_CYTOCHROME_P450	0.146083947	0.028841926	10.99736978	5.80×10^{-27}	1.06×10^{-25}	50.27406765
KEGG_NUCLEOTIDE_EXCISION_REPAIR	-0.147924218	-0.034548422	-10.81772909	3.54×10^{-26}	5.90×10^{-25}	48.48527264
KEGG_HISTIDINE_METABOLISM	0.141129397	0.021983192	10.61525126	2.65×10^{-25}	3.73×10^{-24}	46.49859028
KEGG_PYRIMIDINE_METABOLISM	-0.131300778	-0.022236469	-10.30465471	5.45×10^{-24}	7.12×10^{-23}	43.51254164

KEGG_VALINE_LEUCINE_AND_ISOLEUCINE_DEGRADATION	0.153478623	-0.007471823	10.04483988	6.45×10^{-23}	7.87×10^{-22}	41.07258011
KEGG_BASAL_TRANSCRIPTION_FACTORS	-0.125528537	-0.027725673	-9.929554774	1.90×10^{-22}	2.17×10^{-21}	40.00698728
KEGG_OTHER_GLYCAN_DEGRADATION	0.175800955	0.019427585	9.890367118	2.74×10^{-22}	2.94×10^{-21}	39.64717574
KEGG_P53_SIGNALING_PATHWAY	-0.100263498	-0.022502686	-9.501342592	9.57×10^{-21}	9.73×10^{-20}	36.14194905
KEGG_ONE_CARBON_POOL_BY_FOLATE	-0.152203061	-0.017954202	-9.443422851	1.61×10^{-20}	1.55×10^{-19}	35.63051451
KEGG_CIRCADIAN_RHYTHM_MAMMAL	0.141519034	0.007701052	8.955475119	1.14×10^{-18}	1.04×10^{-17}	31.43077148
KEGG_SPLICEOSOME	-0.116852261	-0.035182057	-8.608683432	2.10×10^{-17}	1.83×10^{-16}	28.56573926
KEGG_TYROSINE_METABOLISM	0.101960565	0.015974197	8.354365901	1.67×10^{-16}	1.27×10^{-15}	26.52887523
KEGG_RNA_DEGRADATION	-0.110027214	-0.031188691	-8.246568899	3.94×10^{-16}	2.89×10^{-15}	25.68205594
KEGG_METABOLISM_OF_XENOBIOTICS_BY_CYTOCHROME_P450	0.110526159	0.023576746	8.131884881	9.76×10^{-16}	6.87×10^{-15}	24.79201236
KEGG_GLYOXYLATE_AND_DICARBOXYLATE_METABOLISM	-0.131759846	-0.015322131	-7.953789533	3.90×10^{-15}	2.46×10^{-14}	23.43218524
KEGG_BUTANOATE_METABOLISM	0.108293295	0.001410922	7.912051019	5.37×10^{-15}	3.28×10^{-14}	23.11744302
KEGG_GLYCOSAMINOGLYCAN_BIOSYNTHESIS_KERATAN_SULFATE	-0.123290563	-0.001048055	-7.841212415	9.23×10^{-15}	5.28×10^{-14}	22.58670561
KEGG_PENTOSE_PHOSPHATE_PATHWAY	-0.106393055	-0.023826327	-7.731243043	2.12×10^{-14}	1.18×10^{-13}	21.77139979
