# Supplementary material for: A Metabolism-Related Gene Prognostic Index for Prediction of Response to Immunotherapy in Lung Adenocarcinoma
Source: Int J Mol Sci. 2022 Oct 12;23(20):12143. doi: 10.3390/ijms232012143 (PMC9602971; doi:10.3390/ijms232012143)
Supplement: Supplementary file 1 [file ijms-23-12143-s001.zip › Table S5.pdf]

Table S5. Detailed information on ICI response analysis in different MRG subgroups

ID	Age	Sex	Immunotherapy drug	PFS event	PFS(years)	MRG score	MRG	Response
GSM2445691	44	Female	Nivolumab	Dead	0.96666667	0.190427049	high	Non-responder
GSM2445692	53	Male	Nivolumab	Alive	12.1	0.182545713	high	Responder
GSM2445699	61	Male	Pembrolizumab	Dead	6.86666667	0.110747342	high	Responder
GSM3589675	47	Male	Nivolumab			0.100025146	high	Responder
GSM2445701	70	Male	Pembrolizumab	Alive	4.9	0.099949091	high	Responder
GSM2445700	42	Male	Pembrolizumab	Dead	3.46666667	0.097634361	high	Responder
GSM3589680	64	Male	Nivolumab			0.09642655	high	Non-responder
GSM3589686	72	Male	Nivolumab			0.089002044	high	Non-responder
GSM3589682	75	Male	Nivolumab			0.07459559	high	Non-responder
GSM2445695	46	Female	Nivolumab	Dead	20.7	0.063246578	high	Responder
GSM3589677	58	Male	Nivolumab			0.062349636	high	Responder
GSM3589676	65	Male	Nivolumab			0.019919146	high	Responder
GSM3589678	55	Female	Nivolumab			0.018237011	high	Non-responder
GSM2445696	61	Male	Pembrolizumab	Dead	18.7	0.015464987	high	Responder
GSM2445702	61	Female	Nivolumab	Dead	2.8	-0.010517452	low	Non-responder
GSM3589688	34	Female	Nivolumab			-0.054191376	low	Responder
GSM2445694	52	Male	Nivolumab	Dead	6.3	-0.054310341	low	Responder
GSM3589684	67	Male	Nivolumab			-0.061575523	low	Non-responder
GSM2445697	66	Male	Pembrolizumab	Dead	0.7	-0.079043605	low	Non-responder
GSM3589683	54	Male	Nivolumab			-0.08253213	low	Responder
GSM3589679	56	Male	Nivolumab			-0.087495488	low	Non-responder
GSM2445693	51	Male	Pembrolizumab	Dead	1.4	-0.092421248	low	Non-responder
GSM3589681	65	Male	Nivolumab			-0.101236124	low	Non-responder
GSM3589674	76	Male	Nivolumab			-0.146002015	low	Non-responder
GSM2445698	47	Female	Pembrolizumab	Dead	0.7	-0.146799107	low	Non-responder
GSM3589687	66	Male	Nivolumab			-0.147966044	low	Non-responder
GSM3589685	61	Male	Nivolumab			-0.215875455	low	Non-responder
GSM3589673	72	Male	Nivolumab			-0.216036899	low	Non-responder
