# Supplementary figures and images for: A Metabolism-Related Gene Prognostic Index for Prediction of Response to Immunotherapy in Lung Adenocarcinoma
Source: Int J Mol Sci. 2022 Oct 12;23(20):12143. doi: 10.3390/ijms232012143 (PMC9602971; doi:10.3390/ijms232012143)

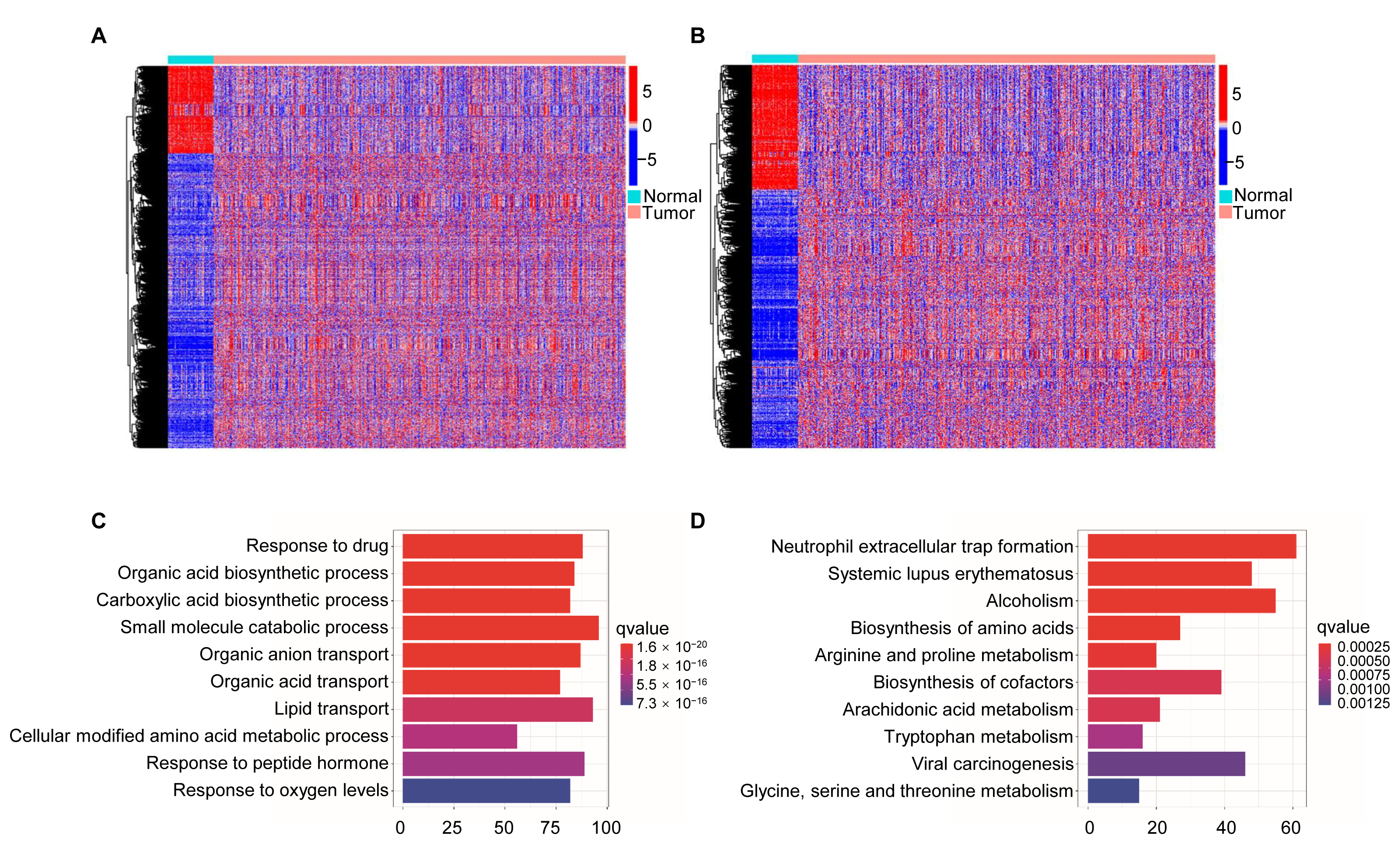

Supplement: Supplementary file 1 [file ijms-23-12143-s001.zip › FigS1.tif]

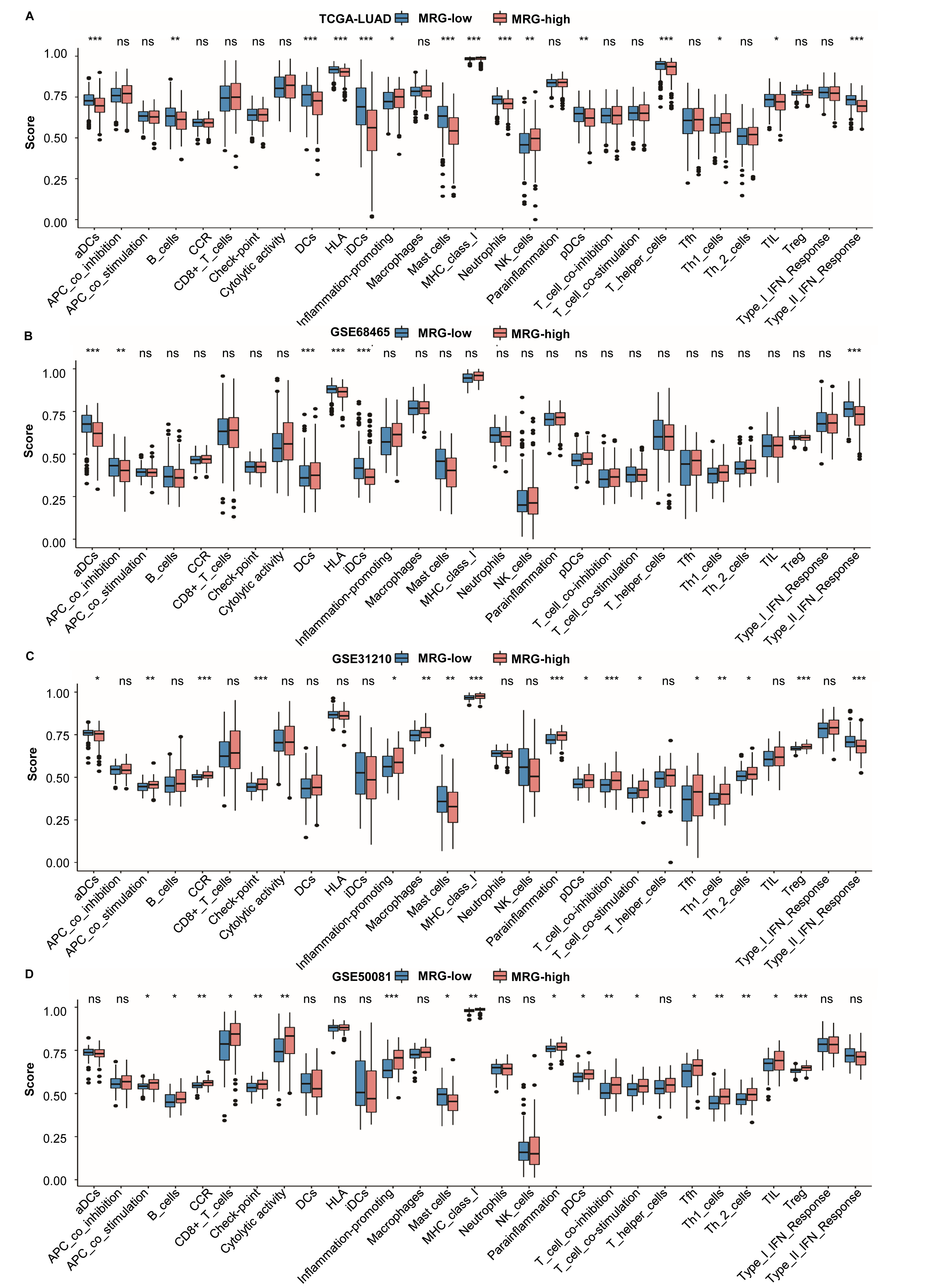

Supplement: Supplementary file 1 [file ijms-23-12143-s001.zip › FigS10.tif]

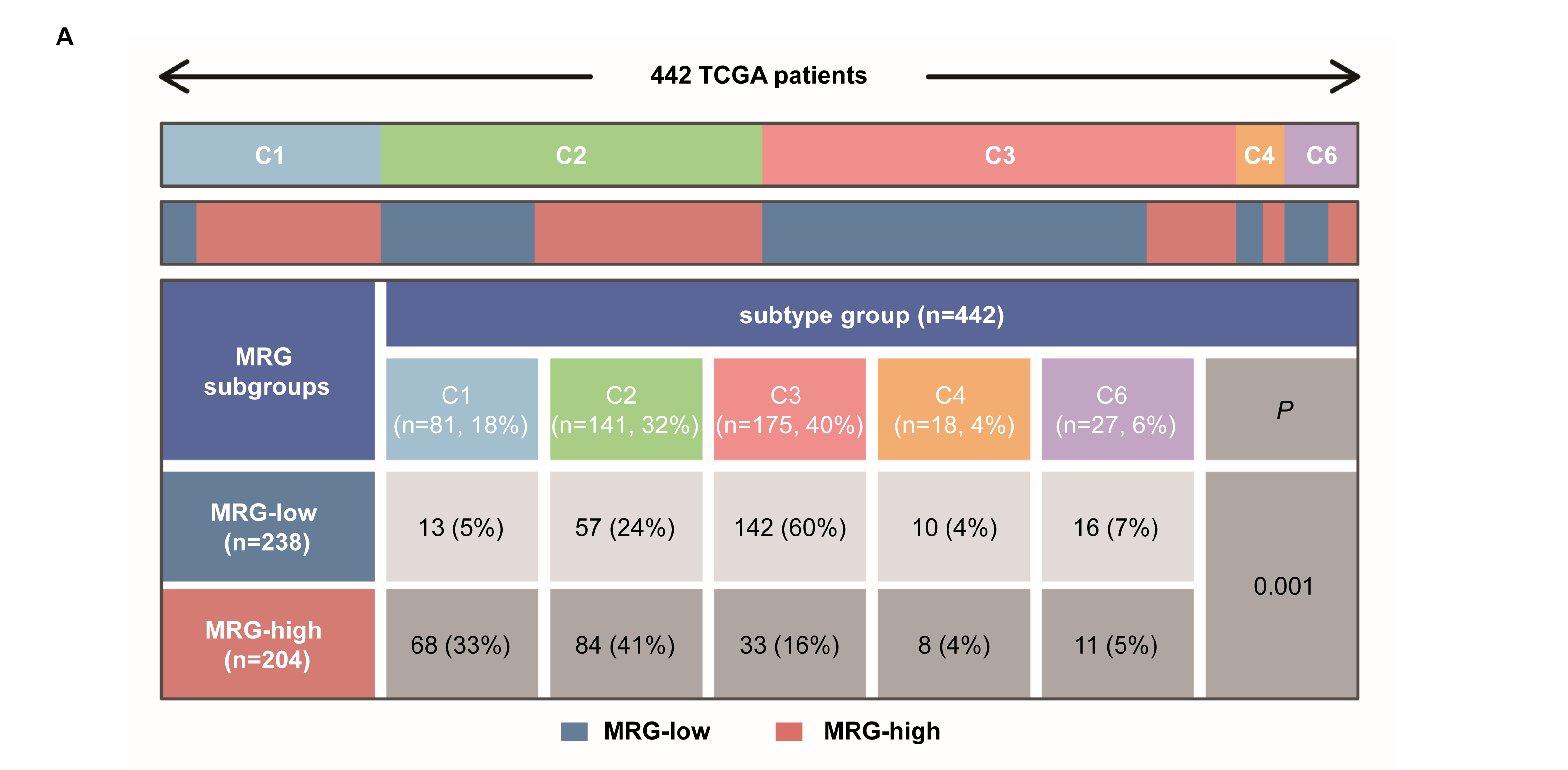

Supplement: Supplementary file 1 [file ijms-23-12143-s001.zip › FigS11.tif]

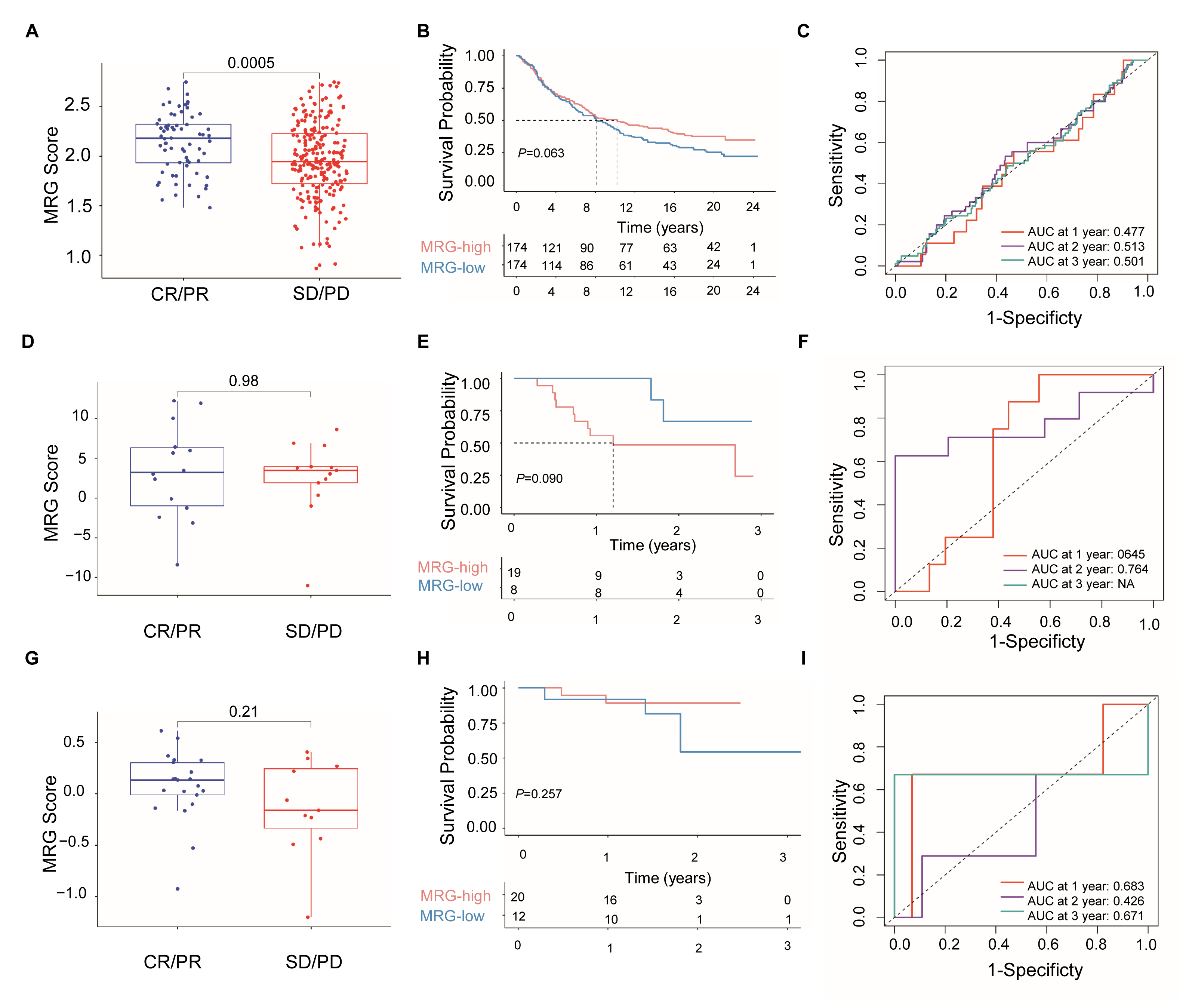

Supplement: Supplementary file 1 [file ijms-23-12143-s001.zip › FigS12.tif]

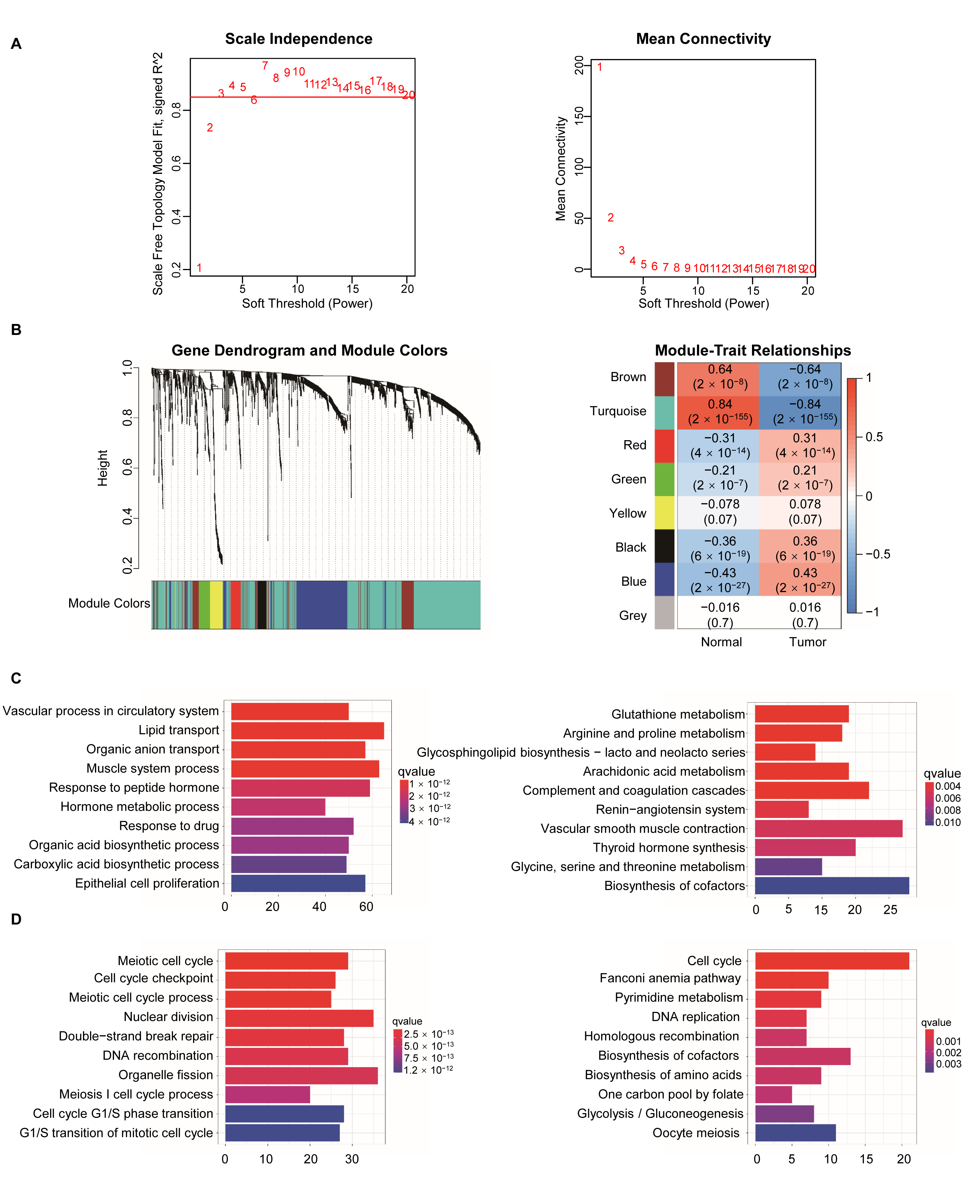

Supplement: Supplementary file 1 [file ijms-23-12143-s001.zip › FigS2.tif]

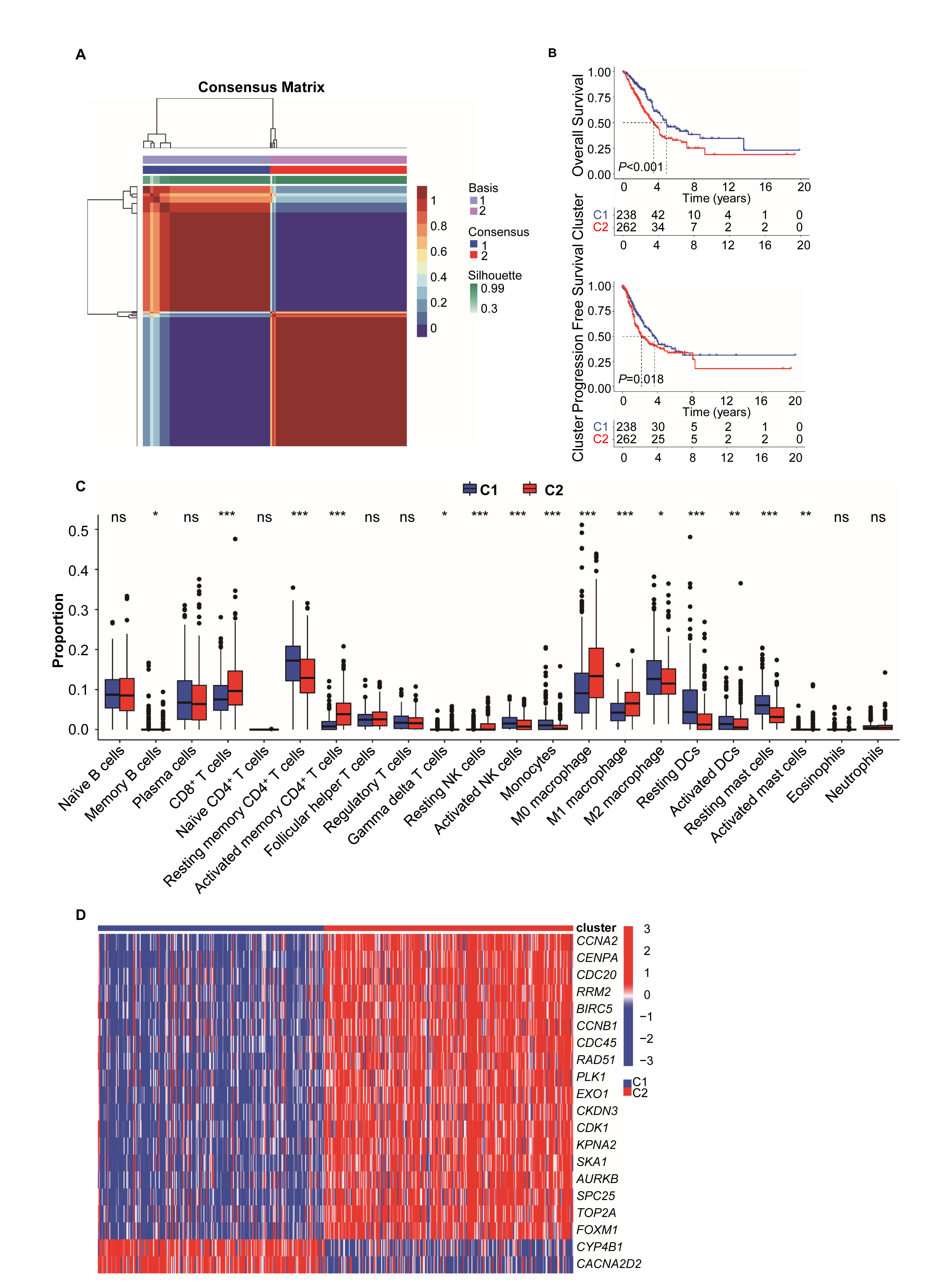

Supplement: Supplementary file 1 [file ijms-23-12143-s001.zip › FigS3.tif]

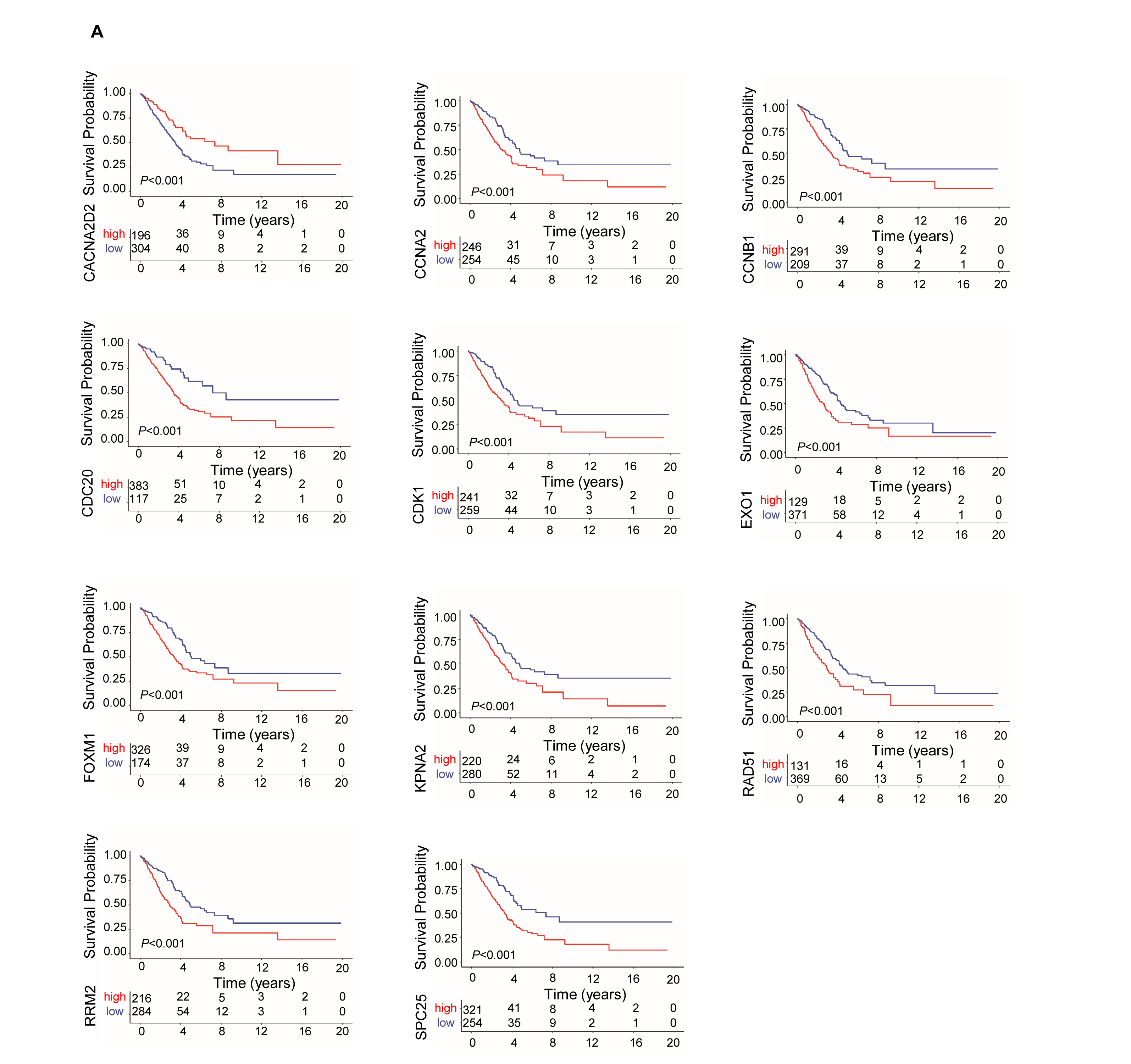

Supplement: Supplementary file 1 [file ijms-23-12143-s001.zip › FigS4.tif]

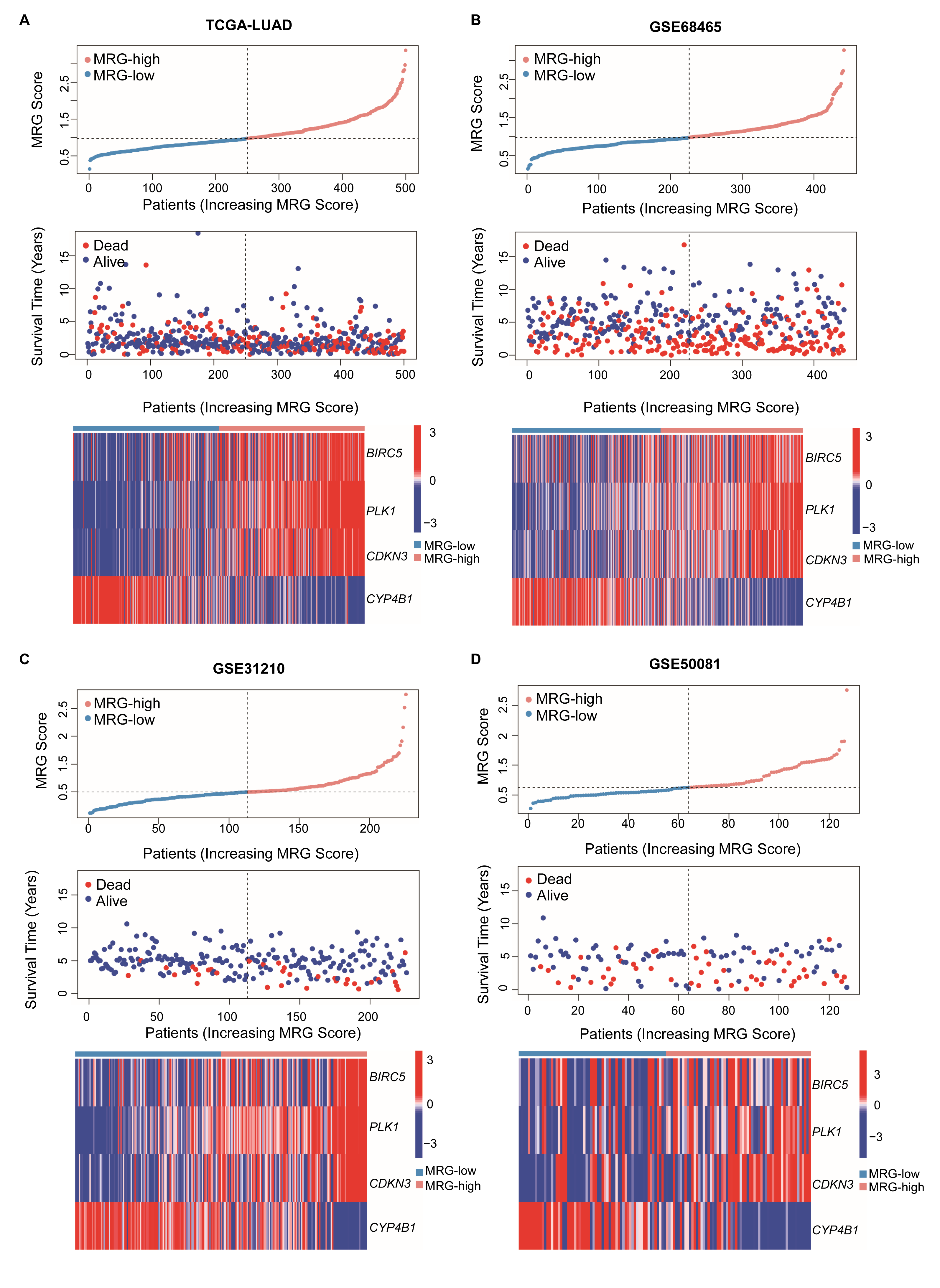

Supplement: Supplementary file 1 [file ijms-23-12143-s001.zip › FigS5.tif]

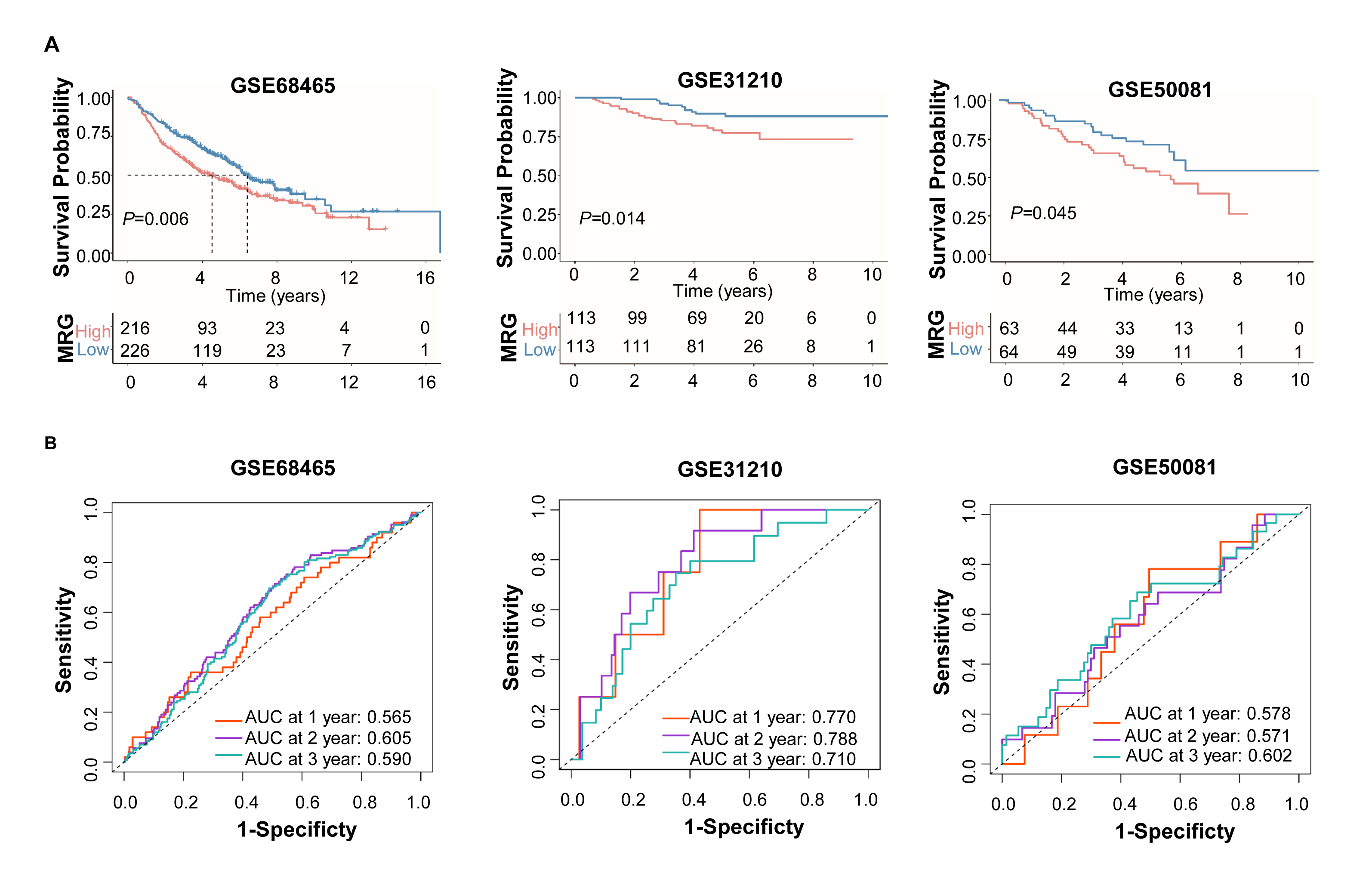

Supplement: Supplementary file 1 [file ijms-23-12143-s001.zip › FigS6.tif]

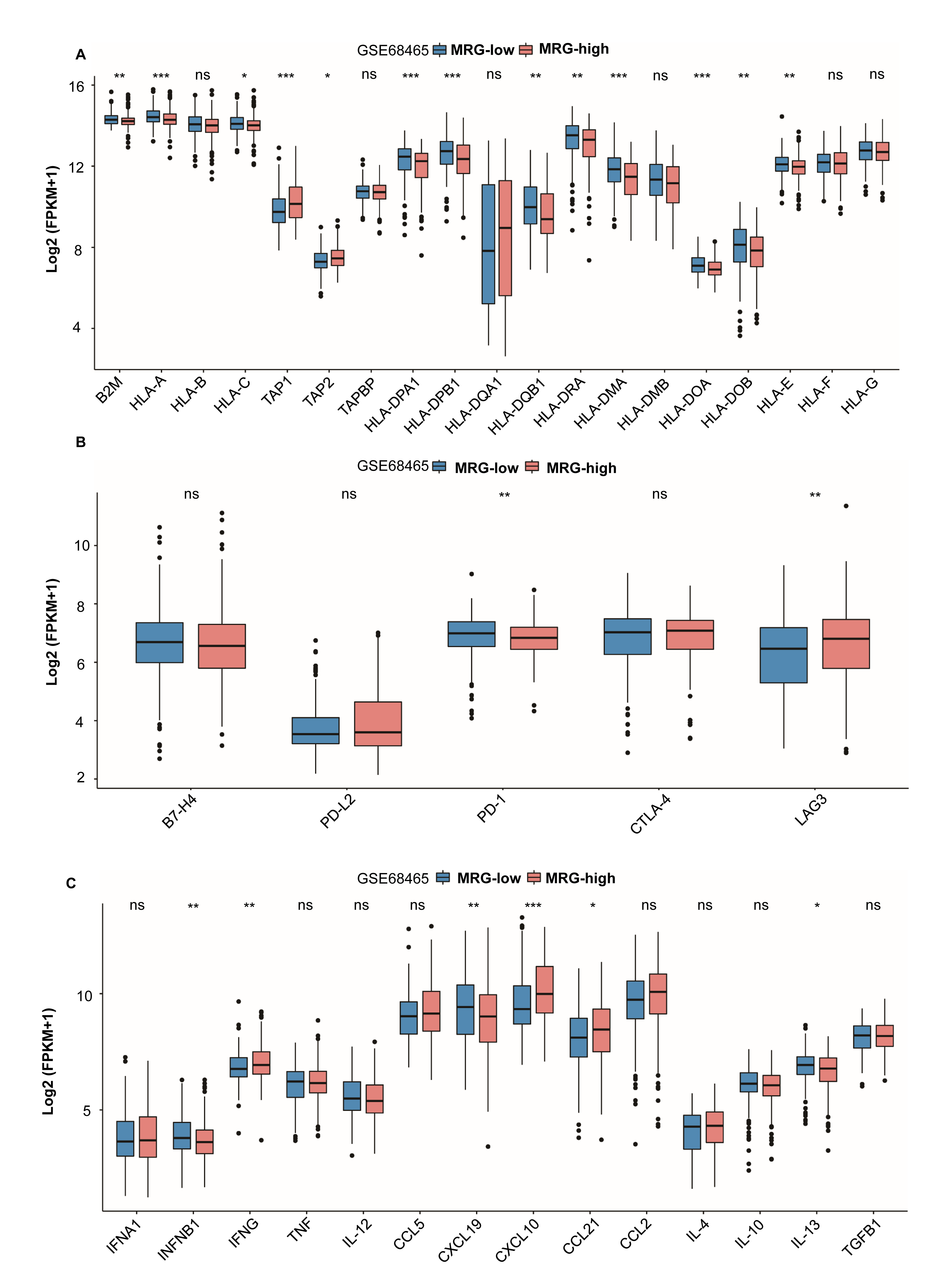

Supplement: Supplementary file 1 [file ijms-23-12143-s001.zip › FigS7.tif]

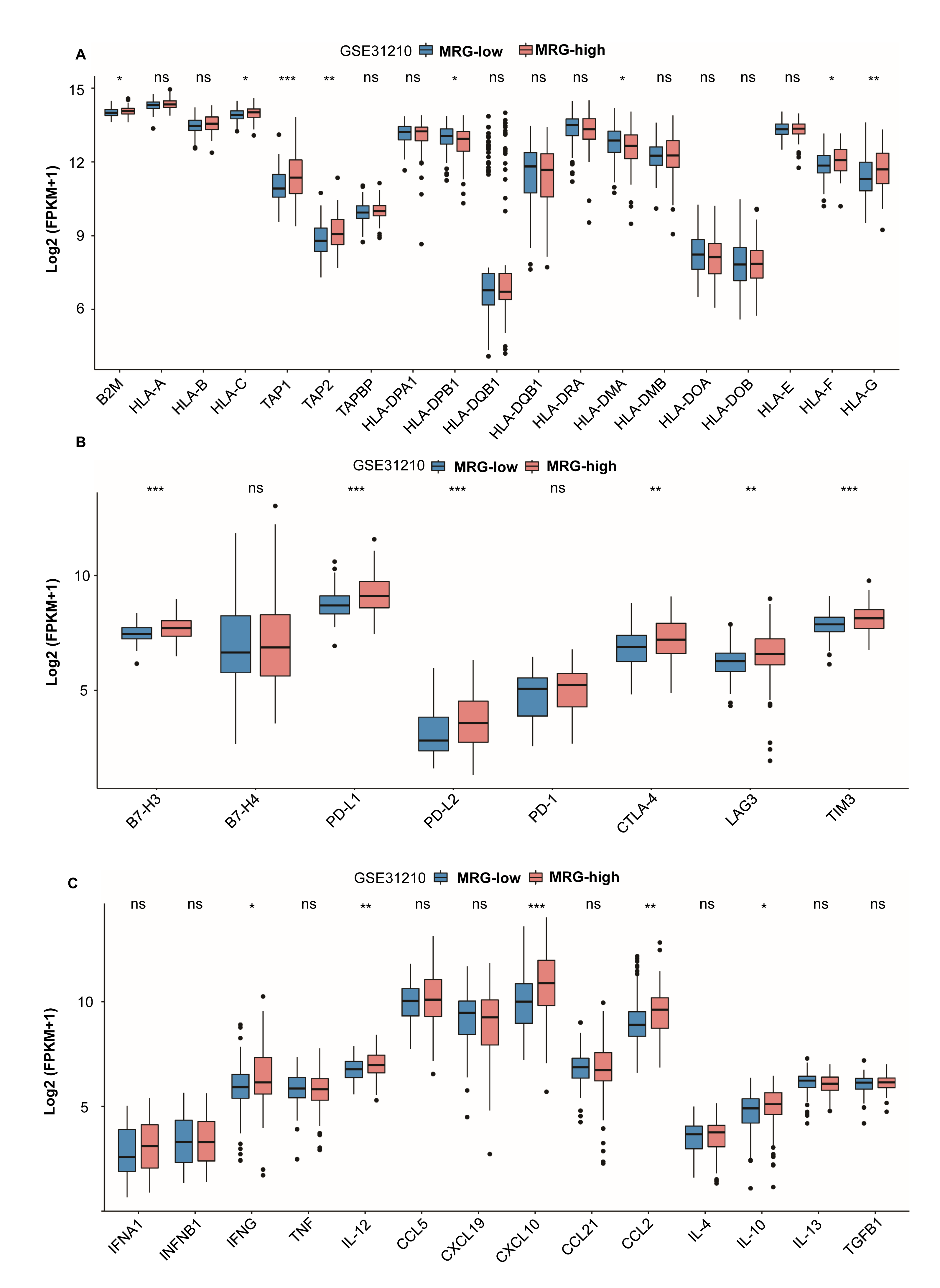

Supplement: Supplementary file 1 [file ijms-23-12143-s001.zip › FigS8.tif]

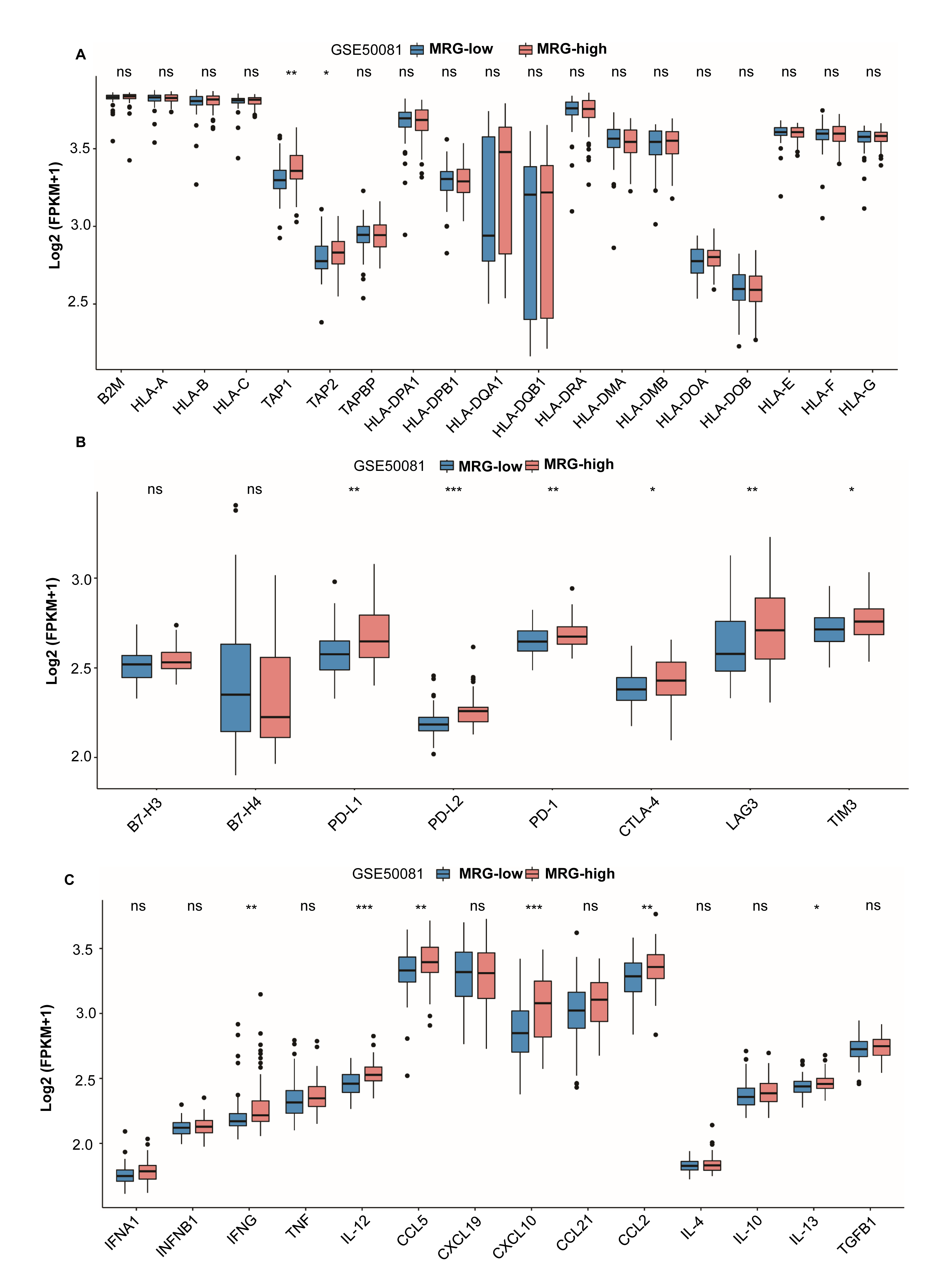

Supplement: Supplementary file 1 [file ijms-23-12143-s001.zip › FigS9.tif]
